# The effect of lemborexant on insomnia in patients with psychiatric disorders: Detailed evaluation using the Athens Insomnia Scale

**DOI:** 10.1002/pcn5.165

**Published:** 2024-01-21

**Authors:** Tomonori Murayama, Yuji Ito, Kenji Narita, Tetsuro Ishida, Shiro Hinotsu, Masahiko Fujita

**Affiliations:** ^1^ Department of Psychiatry Asahikawa Keisenkai Hospital Hokkaido Japan; ^2^ Department of Psychiatry Kushiro Red Cross Hospital Hokkaido Japan; ^3^ Graduate school of Medicine Sapporo Medical University Hokkaido Japan; ^4^ Department of Psychiatry Japan Health Care University Hokkaido Japan; ^5^ Department of Biostatistics and Data Management Sapporo Medical University Hokkaido Japan; ^6^ Wellness Boyo Hospital Otaru Sleep Disorders Clinic Hokkaido Japan

**Keywords:** benzodiazepine hypnotic, insomnia, lemborexant, sleep medicine

## Abstract

**Aim:**

Chronic insomnia disorder is common and associated with reduced quality of life. Benzodiazepine hypnotics are commonly prescribed for insomnia, but have potential side effects such as concentration impairment, somnolence, and dependence. Lemborexant (LEM) is an orexin receptor antagonist considered to have fewer side effects than benzodiazepine hypnotics. This study evaluated the effect of LEM on sleep in detail and examined whether benzodiazepine hypnotics can be gradually tapered by adding LEM.

**Methods:**

We retrospectively examined the effectiveness of LEM in 28 outpatients with insomnia. Insomnia symptoms were assessed using the Athens Insomnia Scale (AIS) before and after LEM administration. We also attempted to taper benzodiazepine hypnotics and assessed benzodiazepine dose using diazepam equivalents for some patients taking benzodiazepine hypnotics. Wilcoxon's signed‐rank test was used for statistical analysis.

**Results:**

The mean AIS score was significantly improved after LEM treatment (8.7 ± 5.2 vs. 3.8 ± 3.3; P < 0.01). Among the AIS subitems, significant improvement was observed for six items: sleep induction, awakenings during the night, sleep quality, well‐being, functioning capacity, and sleepiness during the day. The mean benzodiazepine dose was significantly lower after LEM treatment (4.6 ± 5.0 mg vs. 2.1 ± 3.3 mg; P < 0.01).

**Conclusions:**

This study indicated the potential of LEM for improving insomnia and reducing benzodiazepine dose.

## Graphical abstract

We indicated the potential of lemborexant for improving insomnia and reducing benzodiazepine dose in patients with psychiatric disorders.

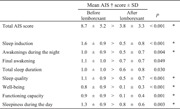

## INTRODUCTION

Chronic insomnia disorder is a common disorder associated with subjective daytime fatigue, low energy, impaired cognitive performance, and reduced quality of life.^1^ Benzodiazepine (BZD) receptor agonists are mainly used as hypnotic, anxiolytic, muscle‐relaxant, and antiepileptic drugs. However, BZD hypnotics have several potential adverse effects, including drowsiness, decline in concentration, memory loss,^2^ and the risk of dependence.^3^ In older patients, the potential for BZD hypnotics to elicit cognitive deficits^4^ and increase the risk of falls^5^ is relatively high, therefore the clinical guidelines of the American Academy of Sleep Medicine suggest that long‐term hypnotic treatment should be indicated only for patients with severe or refractory insomnia or chronic illness.^6^ It has also been suggested that BZD hypnotics should only be used for a short‐term period of up to 4 weeks to prevent the occurrence of difficulties associated with long‐term use.^7^

Despite these adverse effects and the availability of safer and effective alternatives, BZD hypnotics are widely prescribed in the general population.^8^ Although the risks associated with BZD hypnotics are widely recognized, many physicians continue to prescribe BZD hypnotics, often citing patient dependence and benefit as justification.^9^ BZD hypnotics have been reported to be among the most common inappropriate prescriptions in older people,^10^ with a reported prevalence of use ranging from 5% to 32% in community‐dwelling older adults independently of mental health status.^11^, ^12^ Although discontinuation of BZD hypnotics is likely to cause withdrawal symptoms, including rebound insomnia,^13^ discontinuing the long‐term use of BZDs in patients with chronic insomnia is an important issue in the reduction of the risk of these adverse effects. Some strategies for discontinuing BZD hypnotics, including gradual tapering and psychosocial interventions, have been proposed,^6^ for example Japanese guidelines recommend that the amount of BZD hypnotics is reduced by 25% of the dose every 2–4 weeks,^14^ but the most effective approach is unclear.^15^, ^16^, ^17^

In recent years, ramelteon as a melatonin receptor agonist and suvorexant as an orexin receptor antagonist have been developed as substitutes for BZD hypnotics. However, ramelteon does not have sleep‐maintaining effects,^18^ and suvorexant tablets are large and cannot be crushed, making them difficult for older patients to take. Thus, neither of these alternatives provide sufficient sleep treatment.

Lemborexant (LEM) is an antagonist of the orexin receptor, which is thought be involved in the stabilization of wakefulness and has been reported to assist the process of falling asleep and preventing nocturnal awaking. Furthermore, LEM has low dependence potential, is ineffective as a muscle relaxant, and does not significantly affect cognitive function, therefore LEM has potential as an insomnia medication that lacks the problematic side effects of other pharmacological treatment options. There are only a few naturalistic studies conducted by Suzuki et al.^19^, ^20^ in Japan that have evaluated the effects of LEM on patients with insomnia disorder but have not include a detailed evaluation using subitems of Athens Insomnia Scale (AIS).

The primary objective of this study was to investigate in detail the effects of LEM on sleep. The secondary objective was to examine whether the addition of LEM would enable gradual tapering of BZD hypnotics in a subgroup taking BZD hypnotics.

## MATERIALS AND METHODS

We conducted a retrospective search of medical records. Among the patients who regularly visit the psychiatric outpatient department of Kushiro Red Cross Hospital, we selected patients who met the condition of starting LEM administration as a regular medication for the first time between April 2021 and January 2022. In the current study, the patients who started LEM as an occasional medication and the patients who stopped taking prescribed LEM as a regular medication before next visit because their insomnia improved within a few days were excluded. Diagnoses of psychiatric disorders were made by experienced psychiatrists according to the Diagnostic and Statistical Manual of Mental Disorders, Fourth Edition, Text Revision. Diagnosis of chronic insomnia was performed by experienced psychiatrists using the International Classification of Sleep Disorders, Third Edition.^21^ We appropriately estimated and excluded properly sleep disorders such as obstructive sleep apnea, restless legs syndrome, periodic limb disorder, and circadian rhythm sleep–wake disorders.

The following data were collected: participant age, sex, psychiatric diagnosis, and daily dose of BZD hypnotics. The psychiatric symptoms of the patients included in this study were relatively mild to moderate, and patients with severe psychiatric symptoms who required hospitalization were not included. Patients who could not answer our questionnaire because of dementia or mental retardation were also excluded. Forty‐one patients were receiving LEM, but 13 withdrew early and were not included in the statistical analysis. As a result, 28 Japanese patients were recruited. Participants' demographic data are shown in Table 1.

**Table 1:** Participants' demographic data.

	*n* = 28	
Sex (age)	Male: 10 (64.0 ± 13.2)	Female: 18 (51.2 ± 20.1)
Dose of BZD hypnotics	4.6 ± 5.0 mg (*n* = 17, DE)	
Psychiatric disorder		
Schizophrenia	2	
Depression	2	
Dementia	1	
Anxiety	23	

*Note*: We recorded participants' sex, age, dose of BZD hypnotics, and concomitant psychiatric disorders.

Abbreviations: BZD, benzodiazepine; DE, diazepam equivalent.

### Procedure

LEM was administered as a regular medication to participants who still complained of insomnia after being given sleep hygiene instructions.^22^ We evaluated the degree of insomnia before and after the administration of LEM using the Japanese version of the AIS. The AIS is a self‐assessment psychometric comprehensive instrument designed for quantifying multiple aspects of insomnia. This instrument consists of eight items: sleep induction, awakenings during the night, final awakening, total sleep duration, sleep quality, well‐being, functioning capacity, and sleepiness during the day. The AIS was used in the current study because its high consistency, reliability, and validity make it an invaluable tool that is used widely in clinical practice and sleep research.^23^, ^24^ For some patients taking BZD hypnotics, the dosages of multiple BZD hypnotics were also calculated using the daily diazepam equivalent (DE) method^25^ before and after the start of LEM administration. During LEM administration, we implemented gradual tapering of BZD hypnotics after psychosocial interventions in which we explained drug harms and dependency on BZD therapy among participants receiving long‐term BZD therapy and obtained verbal informed consent. After discussion with each participant, one of the following methods was determined to switch from BZD hypnotics to LEM.

1. Add‐on method: LEM was added to the current medication of BZD hypnotics and then BZD hypnotics were gradually tapered off while adjusting LEM as appropriate.
2. Switching method: Tapering of BZD hypnotics and LEM treatment were initiated at the same time, and tapering of BZD was performed while both drugs were adjusted as needed.

**Figure 1:**
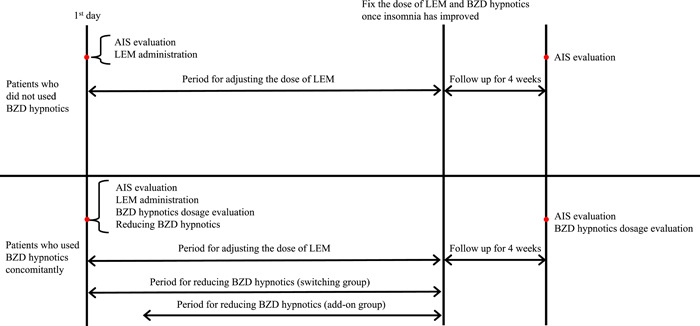
Adjustment schedules for lemborexant (LEM) and benzodiazepine (BZD) hypnotics. AIS, Athens Insomnia Scale

The preintervention AIS assessment and the clinical proposal for LEM treatment and reducing BZD hypnotics were performed on the same day. The daily dose of LEM (and BZD hypnotics in some patients) were adjusted until the patient's sleep improved. In the current study, we used a more gradual approach, with at least a 4‐week observation period before the next dose reduction, considering the patient had been taking BZD hypnotics for many years and the associated risk of rebound insomnia and withdrawal symptoms. Four weeks after the doses of LEM and BZD hypnotics were fixed by titration, the AIS score was assessed. This protocol is shown in Figure 1. Postintervention AIS assessments were not conducted for 13 participants who discontinued LEM therapy. Differences in the AIS score and the total dosage of DE before and after LEM treatment were analyzed using the Wilcoxon signed‐rank test. All calculations were performed with StatFlex (Artech Co., Ltd) version 6 for windows. A level of P < 0.01 was considered to indicate statistical significance. Because this study was not a randomized controlled study and comprised exploratory analyses of a relatively small sample, we performed a per‐protocol analysis rather than an intent‐to‐treat analysis to clarify the efficacy.

The provided data were anonymized. The ethical review board of Kushiro Red Cross Hospital approved this study in accordance with the ethical standards stated in the Declaration of Helsinki (ID: 2021‐2). Because the present study was a retrospective chart review, we used the opt‐out method via the hospital bulletin board, in accordance with the Act on the Protection of Personal Information.

No potential conflicts of interest were disclosed in relation to this study.

## RESULTS

### Participants' characteristics

The demographic data of all participants are shown in Table 1. The number of recruited participants was 28 (male/female = 10/18). The mean age of participants was 55.8 ± 19.6 years. The participants' comorbidities included schizophrenia (*n* = 2), depression (*n* = 2), dementia (*n* = 1), and anxiety (*n* = 23). The average LEM dose was 4.9 ± 1.9 mg. Seventeen of 28 participants (60.7%) had received BZD hypnotics before LEM treatment. The 17 patients were taking between one and three of six different kinds of BZD hypnotics, including zolpidem (*n* = 6), eszopiclone (*n* = 3), brotizolam (*n* = 4), nitrazepam (*n* = 5), flunitrazepam (*n* = 4), and quazepam (*n* = 1), and the mean dosage of BZD hypnotics was 4.6 ± 5.0 mg.

Twelve of the 17 patients that were prescribed BZD hypnotics had taken them for at least 9 years. However, the full details were not available because medical records older than 9 years had been disposed of. The remaining five patients had taken BZD hypnotics for 5.0 ± 4.2 years. The 28 participants had been treated with the following other psychotropic medications that affect sleep: ramelteon (*n* = 5), suvorexant (*n* = 6), trazodone (*n* = 4), mirtazapine (*n* = 2), quetiapine (*n* = 1), chlorpromazine (*n* = 1), levomepromazine (*n* = 1), and BZD anxiolytics (*n* = 11). As the doses of these drugs were not changed during the study observation period, no special statistical adjustment was performed.

### Changes of scale and doses

Table 2 shows the change in AIS score after LEM treatment. The mean AIS scores significantly improved (from 8.7 ± 5.2 to 3.8 ± 3.3; P < 0.01) after treatment. The mean number of days from the start of LEM treatment to post‐intervention AIS assessment was 109.7 ± 53.5. Among the subitems of AIS, significant improvement was observed in six items: sleep induction, awakenings during the night, sleep quality, well‐being, functioning capacity and sleepiness during the day. After LEM treatment daily doses of BZD hypnotics significantly decreased in 17 patients who had received BZD hypnotics previously (from 4.6 ± 5.0 to 2.1 ± 3.3 mg; P < 0.01). Five of these 17 patients were able to quit BZD hypnotics completely and eight patients were able to reduce their doses. Four patients were not able to reduce their doses. It took 119.7 ± 63.0 days to add LEM and attempt to reduce BZD hypnotics in these 17 patients.

**Table 2:** AIS scores before and after lemborexant treatment (*n* = 28)

	Mean AIS score ± SD		
	Before lemborexant	After lemborexant	P
Total AIS score	8.7 ± 5.2	>3.8 ± 3.3	<.001*
Sleep induction	1.6 ± 0.9	>0.5 ± 0.8	<.001*
Awakenings during the night	1.0 ± 0.9	>0.5 ± 0.7	.004*
Final awakening	1.1 ± 1.0	>0.7 ± 0.7	.049
Total sleep duration	1.0 ± 1.0	>0.6 ± 0.8	.030
Sleep quality	1.1 ± 0.9	>0.5 ± 0.7	<.001*
Well‐being	0.8 ± 0.9	>0.1 ± 0.3	<.001*
Functioning capacity	0.9 ± 0.9	>0.1 ± 0.4	.001*
Sleepiness during the day	1.3 ± 0.9	>0.8 ± 0.6	.003*

*Note*: Evaluation of AIS scores before and after lemborexant treatment.

Wilcoxon signed‐rank test *P < 0.01.

Abbreviation: AIS, Athens Insomnia Scale.

In this study, participants discontinued treatment for the following reasons: the participant decided there was a lack of efficacy (*n* = 4), nightmares (*n* = 6), headache (*n* = 2), or dizziness (*n* = 1). All adverse events were transient and completely resolved themselves after discontinuation. These participants were not included in this per‐protocol analysis.

Regarding the method of drug change, of the 28 patients in the continued LEM treatment group, 17 had previously taken BZD hypnotics. The switching method was used for two of these 17 patients. On the other hand, of the 13 patients in the group who discontinued LEM treatment, 11 had previously taken BZD hypnotics. The switching method was used for two of these 11 patients. Two of the four (50.0%) patients who underwent the switching method and nine of the 24 (37.5%) patients who underwent the add‐on method dropped out.

## DISCUSSION

Neurons expressing orexins are distributed within the perifornical lateral hypothalamus and send projections throughout the brain and spinal cord, with particularly dense innervations to nuclei containing monoaminergic and cholinergic neurons constituting the ascending reticular activating system in the brainstem.^26^ Orexin has been shown to increase wakefulness and suppress both non‐REM and REM sleep.^27^

Preclinical studies showed that the orexin 1 receptor (OX1R) and orexin 2 receptor (OX2R) play distinct roles in sleep/wake regulation. Moreover, OX1R has been suggested to suppress REM sleep onset, whereas activation of the OX2R gene regulates the gating of the transition from wakefulness to non‐REM sleep. OX2R plays a major role in preventing the emergence of sleepiness and may be involved in REM sleep control.^28^ A previous study reported that the activation of OX2R, with a lesser contribution from OX1R, suppresses non‐REM sleep, and both OX1R and OX2R are involved in the suppression of REM sleep to a similar degree.^29^ Therefore, if OX2R can be suppressed, REM sleep onset may be enabled and the transition from sleep to the awake state in midnight could be suppressed. It may also be possible to increase non‐REM sleep in this way.

LEM is a dual orexin receptor antagonist that antagonizes both OX1 and OX2 receptors to suppress wakefulness and induce sleep. LEM has unique in vitro effects against orexin receptor subtypes. An in vitro study on receptor selectivity reported that LEM inhibits OX2R more strongly than OX1R, whereas suvorexant has similar inhibitory activity against both OX1R and OX2R.^30^ Considering the mechanisms of the orexin receptor and LEM, an electroencephalography and polysomnography study proposed that LEM administration would be expected to increase REM sleep.^31^

Earlier sleep onset and improved sleep maintenance have been reported in both short‐ and long‐term studies via both objective and subjective evaluations using polysomnography and sleep diaries, respectively.^32^ In addition, a systematic review and network meta‐analysis comparing LEM and suvorexant reported that subjective sleep latency was improved.^33^

In the current study, AIS scores revealed that the items of “sleep induction” and “awakenings during the night” were significantly improved by LEM administration, consistent with the results of previous studies. In particular, the finding that “sleep quality,” “well‐being,” “functioning capacity,” and “sleepiness during the day” significantly improved, even though “final awakening” and “total sleep duration” did not improve suggests that LEM improves overall sleep quality. The mechanism of action of LEM suggests that it may increase REM sleep through suppression of OX2R. Furthermore, we speculate that because LEM does not have the muscle relaxant effects seen in BZD hypnotics, these participants did not feel weakness in the daytime after LEM treatment and gave the answered improved the AIS questionnaire about activity in daytime.

Regarding the method for reducing BZD hypnotics, abrupt discontinuation is likely to cause withdrawal symptoms, including rebound insomnia and anxiety above baseline levels.^34^ Previous studies have suggested that there were no significant effects of alternative pharmacological treatment, including melatonin receptor agonists and orexin receptor antagonists, in preventing rebound insomnia or withdrawal symptoms of BZD hypnotics because they do not act on GABA receptors.^13^ Therefore, to prevent these problems, gradual tapering of BZD hypnotics is recommended. Notably, a previous systematic review indicated that gradual tapering was more effective than conventional approaches for discontinuing BZD hypnotics,^15^ therefore, in the current study, gradual tapering of BZD hypnotics may have prevented rebound insomnia and withdrawal symptoms. On the other hand, the result that the amount of BZD hypnotics could be significantly reduced in the long term may be due to the effect of LEM.

Regarding the method of changing from BZD hypnotics to an orexin antagonist, it should be considered whether the add‐on or the switching method is most appropriate. Hatano et al. reported that the add‐on group exhibited a significantly higher all‐cause discontinuation rate than the switching group.^35^ In addition, intolerability was a significantly stronger risk factor for suvorexant discontinuation in the add‐on group, and the most common adverse effect was oversedation.^35^ However, no one dropped out because of the side effects of oversedation, although we used not only switching but also an add‐on method in this study. This may suggest that LEM has a shorter duration of action than suvorexant.

To the best of our knowledge, no previous reports have compared the effects of LEM before and after administration using the sub‐items of the AIS, and the present study is the first to examine this issue in a clinical setting. This finding has important clinical implications. Here we report that replacement with LEM can safely reduce the dose of BZD hypnotics in patients with psychiatric disorders. LEM does not act via gamma‐aminobutyric acid receptors and is thought to reduce its impact on the electroencephalography spectrum during sleep and act specifically on the sleep–wake cycle, thereby inducing physiological sleep.^36^ The risk of patients developing dependence and tolerance is therefore relatively low.^37^ Similar to the results of previous studies, the treatment interruption rate was relatively low for adverse events in the current study, and no serious adverse events were observed.^30^, ^38^

## LIMITATIONS

Our study involved several limitations. This study was retrospective and did not include a randomized control group for comparison. Thus, prospective randomized controlled trials including patients who were administered LEM and evaluated using AIS are needed to clarify the effects of LEM. This study has the potential selection bias that those patients who had sufficient sleep improvement with BZP hypnotics and did not wish LEM administration were excluded. It is thus possible that LEM would be less effective for patients for whom BZP hypnotic treatment had been successful. Additionally, the number of study participants was small and they were all Japanese. The generalizability of the current findings is limited. We were unable to measure sleep depth with electroencephalography and polysomnography objectively.

## CONCLUSIONS

LEM may significantly improve well‐being, functioning capacity, and sleepiness during the day by improving sleep induction, awakenings during the night, and sleep quality. We suggest that LEM may provide a relatively safe way to reduce the doses of BZD hypnotics relatively safely. Additional studies that include a control group are needed to confirm this conclusion.

## AUTHOR CONTRIBUTIONS

Tomonori Murayama contributed to the design of the study, data analysis, and interpretation of the results and writing the manuscript. Masahiko Fujita contributed to the design of the study and critically reviewed the manuscript. Yuji Ito, Kenji Narita, and Tetsuro Ishida contributed to the analysis of the data. Shiro Hinotsu provided statistical advice. All authors have seen and approved the manuscript.

## CONFLICT OF INTERESTS STATEMENT

The authors declare no conflict of interest. The first author, Tomonori Murayama, currently works at Asahikawa Keisenkai Hospital. Co‐author Kenji Narita is currently affiliated with the Graduate School of Medicine, Sapporo Medical University.

## ETHICS APPROVAL STATEMENT

This study was approved by the Ethics Committee of Kushiro Red Cross Hospital (Permission Number 2021‐2). This study did not include off‐label or investigational use of drugs or clinical trials.

## PATIENT CONSENT STATEMENT

We used the opt‐out method via the hospital bulletin board, in accordance with the Act on the Protection of Personal Information.

## CLINICAL TRIAL REGISTRATION

N/A

## Data Availability

The data that support the findings of this study are available from the corresponding author upon reasonable request.
